# Low Overnight Temperature-Induced Gibberellin Accumulation Increases Locule Number in Tomato

**DOI:** 10.3390/ijms20123042

**Published:** 2019-06-21

**Authors:** Yanbing Li, Meihua Sun, Hengzuo Xiang, Yudong Liu, Hui Li, Mingfang Qi, Tianlai Li

**Affiliations:** 1 College of Horticulture, Shenyang Agricultural University, National & Local Joint Engineering Research Center of Northern Horticultural Facilities Design & Application Technology (Liaoning), Key Laboratory of Protected Horticulture of Education Ministry and Liaoning Province, Shenyang 110866, China; liyanbing09@126.com (Y.L.); dabao8308@163.com (M.S.); xianghengzuo8308@163.com (H.X.); lyd-forever@163.com (Y.L.); 2 Key Laboratory of Agricultural Biotechnology of Liaoning Province, Shenyang 110866, China; 18842585102@163.com

**Keywords:** low temperature, gibberellins, locule number, tomato, RNA-seq

## Abstract

The number of locules in tomato affects fruit size, shape, and the incidence of malformation. Low temperature increases locule number and the incidences of malformation in tomato plants. In this study, three flower bud developmental stages (pre-flower bud differentiation, sepal and petal primordium formation, and carpel primordium formation) under different night temperatures (10, 15, and 20 °C) were used to analyze the reason behind locule number change using an RNA sequencing (RNA-seq) approach, Quantitative real-time PCR (qRT-PCR), and ultra-performance liquid chromatography–tandem mass spectrometry (UPLC-MS). The results showed that the “plant hormone signal transduction”, “starch and sucrose metabolism”, and “diterpenoid biosynthesis” categories were remarkably activated during flower bud differentiation. Transcripts of gibberellin (GA)-related genes and endogenous levels of GAs were analyzed, and it was discovered that *SlGA2ox* genes were significantly downregulated and bioactive GA_1_ and GA_4_ accumulated at lower overnight temperature. Exogenous application of bioactive GA_1_, GA_4_, and PAC (paclobutrazol) showed that GA_1_ and GA_4_ increased the locule number, while PAC decreased the locule number. Taken together, our results suggest that lower overnight temperature reduced the expression of *SlGA2ox* genes, leading to GA_1_ and GA_4_ accumulation, thereby increasing locule number in tomato.

## 1. Introduction

Cold stress, including chilling (0–15 °C) and freezing (<0 °C) temperatures, is a major adverse environmental condition [1,2]. All organisms evolved to cope with cold stress, ensuring the optimal combination of proliferation and survival [3,4,5,6]. Temperature has a profound influence on plant development, as flower and fruit development are highly sensitive to low, nonfreezing temperatures. When plants are exposed to low temperatures, they produce flowers that show alterations in the number, morphology, and pattern of fusion of floral organs [7,8,9]. Consequently, abnormal fruits of low economic value are produced from these flowers. However, the effect of low temperature on shoot apical meristem (SAM) activity and behavior remain poorly understood [9,10]. SAM is vital for the development and growth of multicellular organisms [11,12]. In the dicot plant tomato, the SAM consists of a pool of stem cells which continuously provides cells for new tissue formation (leaves and floral organs) [13]. The tomato locule develops from the carpel, at the inner whorl of the tomato floral organs, and the number of carpels in a tomato flower determines the final number of locules in a mature fruit [14,15]. The number of tomato locules evolved from just 2 to greater than 10, which affects the fruit shape and size; the higher the ovary locule number is, the larger the fruit and the higher the incidence of fruit malformation will be [16,17,18,19].

Cultivated tomato plants produce fruits as much as 1000 times larger than those of their wild progenitors, and fruit size is the primary desired characteristic of commercial tomato varieties and an essential goal for tomato domestication [14,17,18,20]. In this dramatic transition, both carpel cell division and carpel number determine the final size of a tomato fruit [17]. The locule number of tomato was traced to two quantitative trait loci, referred to as *locule-number* (*lc*) and *fasciated* (*fas*) [18,21,22]. The *lc* mutation caused by two single-nucleotide polymorphisms at 1080 bp from the 3′ end of *SlWUSCHEL* (*SlWUS*), is responsible for this increase in locule number [16]. *SlWUS*, which is a candidate gene for *lc*, was proposed for use as a regulator of the number of tomato floral organs and fruit locules [23]. The increase in the number of locules caused by *fas* mutation is due to a 294-kb inversion with breakpoints in intron 1 of *YABBY* and 1 kb upstream of *SlCLAVATA3* (*SlCLV3*) [24]. It was confirmed that a regulatory change in *SlCLV3* underlies the *fas* mutant phenotype [20,24].

The number of locules in tomato is regulated not just by local signals from within the SAM, but also by systemic signals from outside the tissue [19,25,26]. For example, early reports demonstrated that low temperatures could cause the malformation of floral organs, especially petals, stamens, and carpels [7,8,9,27,28]. In tomato, lower temperatures can cause flower malformation, accompanied by an increase in the number of stamens and carpels [7]. Exogenous application of GA_3_ and PAC (paclobutrazol; an inhibitor of gibberellins biosynthesis) can induce an increase or reduction in the number of carpels; this effect was much more obvious for plants grown at lower temperatures [25,26]. However, what remains obscure is the effect of temperature on the regulation level of gibberellins in the SAM.

Tomato seedlings exposed to lower overnight temperature usually develop a large number of malformed flowers and fruit [7,26]. Currently, little is known about the change process of cold acclimation in the SAM, despite the fact that optimum shoot apical development and function are essential for bolstering plant growth and crop productivity under climate change. In this study, we utilized RNA sequencing (RNA-seq) to show that both *C19-GA2ox* and *C20-GA2ox* genes were downregulated at lower overnight temperature. We also found that GA_1_ and GA_4_ accumulated at lower overnight temperature. In addition, the application of GA_1_ and GA_4_ exogenously showed that GA_1_ and GA_4_ increased and PAC decreased the locule number. Our work reveals that lower overnight temperature reduced the expression of *SlGA2ox* genes, leading to GA_1_ and GA_4_ accumulation, thereby increasing the locule number of tomato.

## 2. Results

### 2.1. Phenotypic Analysis of Tomato Fruit at Different Night Temperatures

**Figure 1:**
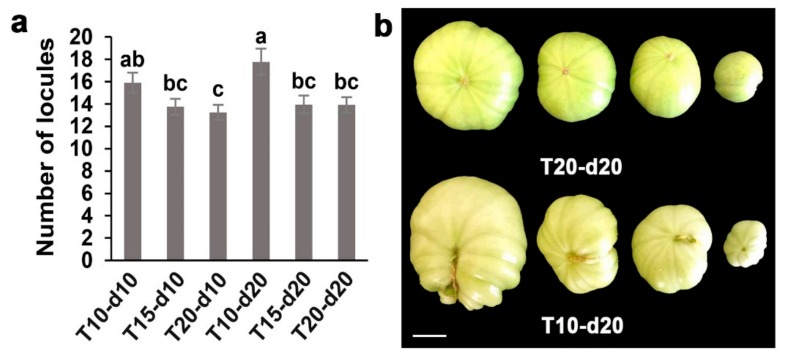
Effects of different overnight temperatures on fruit morphometrical of tomato. (**a**) The effect of different overnight temperatures (T10, T15, and T20) and treatment days (10 and 20 days) on the number of locules. (**b**) The effect of T10 and T20 overnight temperature treatment for 20 days on fruit malformation. The error bars represent the standard errors. Different lowercase letters represent significant differences (*p* < 0.05, Duncan’s multiple range test). Scale bar: 1 cm.

Green-ripe stage fruits of the first inflorescence were used to investigate the locule number. After 10 days of lower overnight temperature, the average number of locules at T10-d10 (“T” stands for different night temperatures and “d” for treatment days) was higher than that at T15-d10 and T20-d10, but only the difference between T10-d10 and T20-d10 treatments was statistically significant. After 20 days of lower overnight temperature, the average number of locules at T10-d20 was 17.78, which was significantly higher than the averages at T15-d20 (13.95) and T20-d20 (13.90). (Figure 1a; Table S1). We noticed that fruit malformation at T10-d20 was more severe than that at T20-d20 after 20 days of treatment (Figure 1b). These results imply that lower overnight temperature increase the locule number and the incidence of fruit malformation.

### 2.2. Overview of Messenger RNA (mRNA) Sequencing Data

In order to determine the effect of lower temperature on the alteration in gene expression during flower bud differentiation, we generated complementary DNA (cDNA) libraries composed of the samples collected from three developmental stages (pre-flower bud differentiation, sepal and petal primordium formation, and carpel primordium formation) at different overnight temperatures with two biological replicates. In total, the numbers of raw reads at CK (control, pre-flower bud differentiation), T10-d10, T15-d10, T20-d10, T10-d20, T15-d20, and T20-d20 reached 73,023,120; 64,078,977; 53,299,090; 52,736,947; 59,988,215; 53,972,546; and 67,265,972, respectively. After removing low-quality reads, we recorded a total of 72,347,574; 63,600,058; 52,897,547; 52,392,032; 59,600,279; 53,397,506; and 66,417,818 reads, which were then mapped to the tomato reference genome using HISAT. Lastly, the numbers of high-quality reads generated from the seven samples of uniquely mapped reads were 65,060,425 (89.93%), 57,752,715 (90.81%), 47,913,998 (90.58%), 47,523,527 (90.71%), 54,114,303 (90.80%), 47,801,791 (89.52%), and 59,583,787 (89.71%) at CK, T10-d10, T15-d10, T20-d10, T10-d20, T15-d20, and T20-d20, respectively (Table 1).

### 2.3. Overall Identification and Functional Annotation of Differentially Expressed Genes (DEGs)

**Figure 2:**
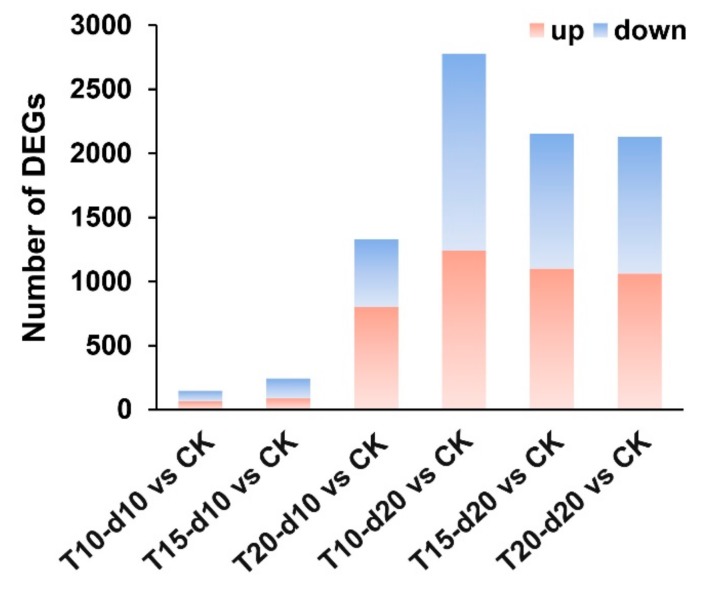
Stat chart of differentially expressed genes (DEGs) in different treatments. Distribution of DEGs on different days at different overnight temperatures. The DEGs were identified with the criteria (log2 (Ratio) ≥1 and *p* < 0.05).

We analyzed the DEGs of seven samples in the RNA-seq datasets. For the 10-day treatment, we observed 63 upregulated and 85 downregulated genes in T10-d10 vs. CK, 86 upregulated and 157 downregulated genes in T15-d10 vs. CK, and 802 upregulated and 525 downregulated genes in T20-d10 vs. CK. For the 20-day treatment, we found 1242 upregulated and 1534 downregulated genes in T10-d20 vs. CK, 1100 upregulated and 1052 downregulated genes in T15-d20 vs. CK, and 1062 upregulated and 1066 downregulated genes in T20-d20 vs. CK (Figure 2; Table S2).

**Figure 3:**
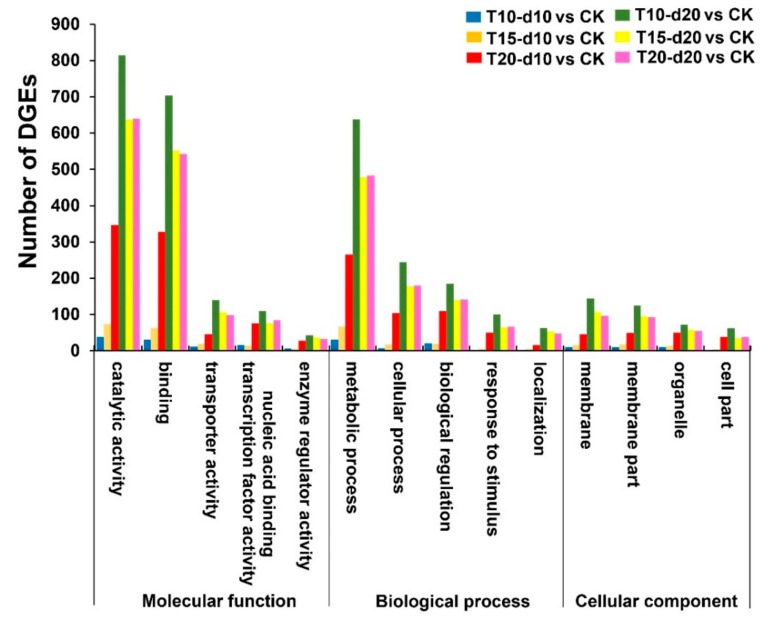
Gene Ontology analysis of DEGs in different treatments. DEGs were annotated in three main categories: biological process, cellular component, and molecular function.

The functional enrichment analysis of the DEGs revealed that the subcategories 21, 22, 29, 29, 28, and 29 were enriched in T10-d10 vs. CK, T15-d10 vs. CK, T20-d10 vs. CK, T10-d20 vs. CK, T15-d20 vs. CK, and T20-d20 vs. CK respectively (Table S3). We classified DEGs into three main categories: “molecular function”, “biological process”, and “cellular component” for analysis. In the “molecular function” category, “catalytic activity”, “binding”, “transporter activity”, “nucleic acid binding transcription factor activity”, and “enzyme regulator activity” were significantly overrepresented. Moreover, in the “biological process” category, “metabolic process”, “cellular process”, “biological regulation”, “response to stimulus”, and “localization” were significantly overrepresented. In the “cellular component” category, “membrane”, “membrane part”, “organelle”, and “cell part” were significantly overrepresented (Figure 3).

We evaluated the biological significance of the DEGs during tomato flower bud differentiation at different night temperatures obtained through pathway-based analysis by using the KEGG database. The pathways of the KEGG categories 18, 26, 17, 21, 30, and 18 were available at T10-d10 vs. CK, T15-d10 vs. CK, T20-d10 vs. CK, T10-d20 vs. CK, T15-d20 vs. CK, and T20-d20 vs. CK, respectively (Table S4). The main pathway with DEG enrichment was “plant hormone signal transduction”, followed by “starch and sucrose metabolism”, “plant-pathogen interaction”, “phenylalanine metabolism”, and “diterpenoid biosynthesis” (Table 2).

### 2.4. Expression Patterns and Endogenous Levels of Gibberellins

According to the results of DEGs enrichment in the KEGG database, the main pathways for DEGs enrichment included “plant hormone signal transduction” and “diterpenoid biosynthesis”. We further analyzed the expression patterns and endogenous levels of gibberellins (GAs). GA biosynthesis starts with *trans*-geranylgeranyl diphosphate (GGDP), which is converted to GA_12_ by four enzymes, namely ent-copalyl diphosphate synthase (CPS), ent-kaurene synthase (KS), ent-kaurene oxidase (KO) and ent-kaurenoic acid oxidase (KAO). Subsequently, GA-20 oxidase (GA20ox) catalyzes the conversion of GA_12_/GA_53_ to the immediate bioactive GA precursors GA_9_/GA_20_, which are further catalyzed by GA-3 oxidase (GA3ox) to the bioactive GAs (GA_1_, GA_3_, GA_4_, and GA_7_) [29]. The enzymes responsible for inactivating GAs are GA-2 oxidases (GA2ox), including C19-GA2oxs and C20-GA2oxs, the substrates of C19-GA2oxs, C19-GAs (GA_1_ and GA_4_), and their precursors (GA_20_ and GA_9_) can be converted to inactive GAs (GA_8_, GA_34_, GA_29_, and GA_51_). In contrast, the substrates of C20-GA2oxs, which are C20-GAs (GA_12_ and GA_53_), can be hydroxylated to the inactive forms GA_110_ and GA_97_, respectively [30,31,32].

**Figure 4:**
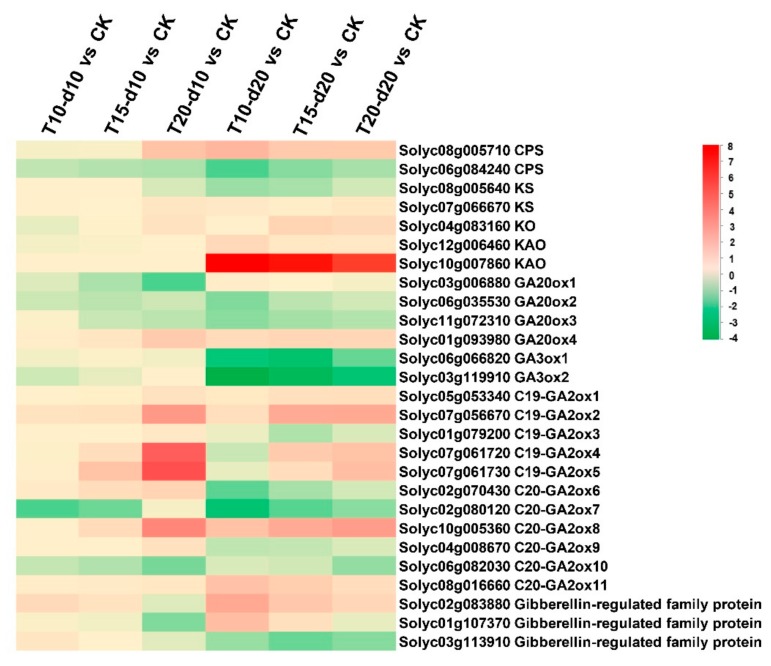
Gene expression pattern of the gibberellin (GA)-related genes in different treatments. Green indicates a decrease and red an increase in gene expression (see color set scale on top right corner). Detailed information on each gene and its expression level is listed in Table S5.

**Figure 5:**
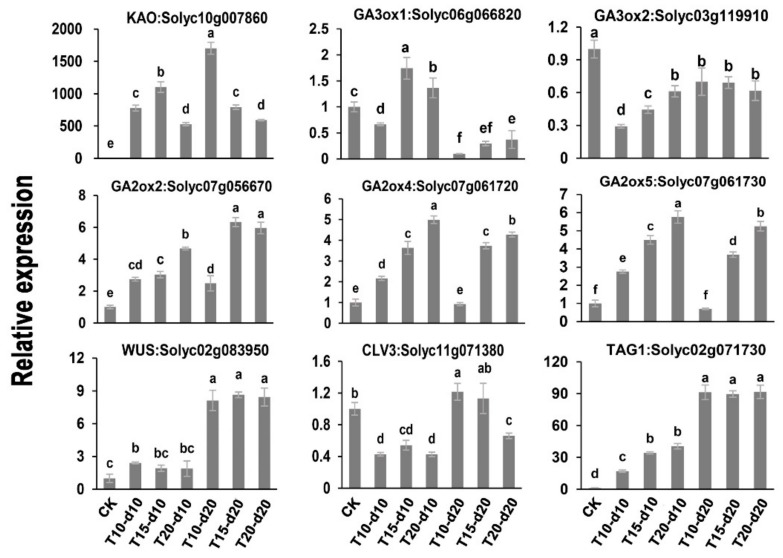
qRT-PCR analysis of *KAO*, *GA3ox1*, *GA3ox2*, *GA2ox2*, *GA2ox4*, *GA2ox5*, *WUS*, *CLV3*, and *TAG1* in the shoot apex at CK (as control), T10-d10, T15-d10, T20-d10, T10-d20, T15-d20, and T20-d20 to confirm RNA sequencing (RNA-seq) data. The data are the mean values corresponding to three independent experiments. The error bars represent the standard errors. Different lowercase letters represent significant differences (*p* < 0.05, Duncan’s multiple range test).

Figure 4 depicts the effects of the various temperature regimes involved in biosynthesis and responsive genes of GAs. The expression level and fold change of GA-related genes are shown in Table S5. The results show that the expression of *KAO* was upregulated at T10-d10 and T10-d20 with respect to T20-d10 andT20-d20, respectively (Figure 5). In agreement with this finding, the GA_53_ content showed accumulation at T10-d10 and T10-d20 with respect to T20-d10 and T20-d20, respectively (Table 3). After treatment for 10 days at different overnight temperatures, *GA20ox1* and *GA20ox3* were slightly upregulated and *GA20ox4* was slightly downregulated at lower overnight temperature, and, after treatment for 20 days at different overnight temperatures, *GA20ox1* was slightly upregulated and *GA20ox2* was slightly downregulated at lower overnight temperature, but their expression levels were not very sensitive to lower overnight temperature (Figure 4). After treatment for 10 days at different overnight temperatures, *GA3ox1* and *GA3ox2* were downregulated at lower temperatures (Figure 4). The content of GA_4_, the catalytic product of GA-3 oxidase, decreased at lower overnight temperature after treatment for 10 days (Table 3). After treatment for 20 days at different overnight temperatures, *GA3ox1* and *GA3ox2* were insensitive to overnight temperature, and the expression of *GA3ox* genes was downregulated with prolonged treatment time (Figure 4 and Figure 5). As a result, the content of active GA_3_ and GA_7_ decreased during flower bud differentiation, GA_3_ was only detected at the pre-floral bud differentiation stage, and there was no significant difference in GA_7_ content after lower overnight temperature treatment (Table 3). It is worth noting that lower overnight temperature inhibited the expression of *C19-GA2ox* genes (*GA2ox2*, *GA2ox4*, and *GA2ox5*) and *C20-GA2ox* genes (*GA2ox6*, *GA2ox7*, and *GA2ox8*) (Figure 4), and active GA_1_ and GA_4_ and their precursors GA_20_ and GA_9_ accumulated at lower overnight temperature, whereas GA-2 oxidase is not responsible for the catabolism of active GA_3_ and GA_7_. We found that GA_7_ is not sensitive to different overnight temperatures at 10 days or 20 days (Table 3).

In addition, we analyzed the expression of GA responsive genes and found that GA-regulated genes were upregulated at lower overnight temperature (Figure 4). This further explains the increase in GA_1_ and GA_4_ content at lower overnight temperature. In conclusion, we speculated that the increase in locule number at lower overnight temperature was mainly due to the downregulation of *SlGA2ox* genes, which resulted in the accumulation of active GA_1_ and GA_4_.

### 2.5. Validation of DEGs by qRT-PCR

To verify the precision and reproducibility of the transcriptome data, *KAO*, *GA3ox1*, *GA3ox2*, *GA2ox2*, *GA2ox4*, *GA2ox5*, *WUS*, *CLV3*, and *TAG1* in the shoot apex were selected for qRT-PCR analysis at CK (as control), T10-d10, T15-d10, T20-d10, T10-d20, T15-d20, and T20-d20 to confirm RNA-seq data. The qRT-PCR assay results were consistent with the transcriptional data (Figure 5; Table S6).

### 2.6. Exogenous Gibberellin Applications Changed the Number of Tomato Locules

**Figure 6:**
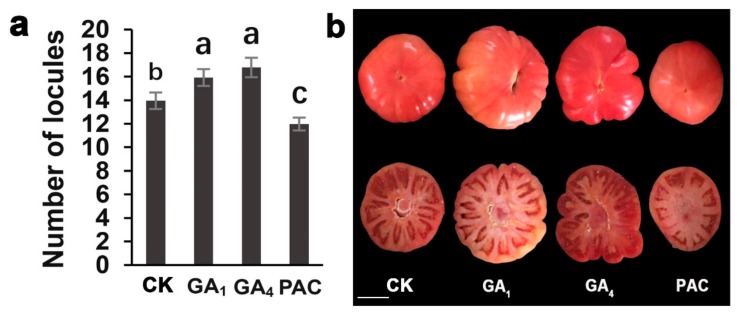
Fruit morphology of application of GA_1_, GA_4_, paclobutrazol (PAC), and H_2_O, as a control. (**a**) Quantification and comparison of locule numbers of GA_1_ (100 μM), GA_4_ (100 μM), PAC (100 μM), and H_2_O treatments. (**b**) The effect of GA_1_, GA_4_, PAC, and H_2_O treatments on locule number and fruit morphology. The error bars represent the standard errors. Different lowercase letters represent significant differences (*p* < 0.05, Duncan’s multiple range test). Scale bar: 3 cm.

To investigate if this observed higher GA_1_ and GA_4_ accumulation at lower overnight temperature is actually necessary for regulating the locule number of tomatoes, we investigated locule numbers following exogenous administration of GA_1_, GA_4_, PAC, and H_2_O. The locule number significantly increased in tomato plants that were treated with exogenous GA_1_ and GA_4_ applications. In contrast, PAC significantly reduced the locule number in tomato (Figure 6; Table S1).

## 3. Discussion

### 3.1. Low Temperature Induced Multi-Locule Fruit Formation

More than 80% of the Earth’s biosphere is permanently or seasonally subjected to temperatures below 5 °C, which has a significant impact on reproductive success and fitness. Plants must cope with low temperatures on a daily or seasonal basis. Worsening global warming is the cause of the frequent occurrence of colder and longer winters in northern mid-latitudes [33,34,35]. The SAM is responsible for the development of all post-embryonic aerial organs, such as the leaves, stems, and floral organs [12,36,37]. Plants grown under suboptimal growth temperature conditions during their reproductive development show a prominent change in the number of reproductive floral organs [7,8,26,38]. Our previous research indicated that overnight temperature treatment at 6 °C for 30 days increased the incidence of fruit malformation of the first inflorescence to over 60%. Moreover, the incidence of fruit malformation at 12 °C was lower, at 28.45%. Therefore, we selected night temperatures of 10 °C, 15 °C, and 20 °C to explore the molecular mechanisms of low night temperature regulation of tomato locule number. Our findings confirm that a lower overnight temperature of 10 °C increased the locule number and incidence of fruit malformation in tomato (Figure 1).

To date, a few loci are known to regulate the number of tomato locules: the mutation of the *fas* or *lc* loci to change *SlCLV3* or *SlWUS* expression [16,24]. In our RNA-seq and qRT-PCR findings, *SlWUS* expression gradually increased during the process of flower bud differentiation, but was not sensitive to lower overnight temperature (Figure 5; Tables S5 and S6). *SlClV3* is a negative regulator of tomato locule number [24]. Conversely, its expression is slightly increased under lower overnight temperature at 20 days (Figure 5; Table S6). Therefore, we speculated that lower overnight temperature did not affect the locule number of tomatoes via *WUSCHEL-CLAVATA3* meristem size regulators, in agreement with previous studies [7]. In addition, as previously reported, low temperature increased the number of flower organs by inducing *SlTAG1* expression [7]. However, in our RNA-seq findings, *SlTAG1* expression gradually increased with flower bud differentiation, and its expression level was lower at T10-d10 than at T15-d10 and T20-d10 at 10 days; there was no significant difference at different overnight temperatures at 20 days (Figure 5; Table S6,). So far, research into the function of *SlTAG1* revealed that *SlTAG1* does not alter the number of carpels, but affects stamen development and fruit ripening [39,40]. Therefore, our results indicate that lower overnight temperature increased the number of tomato locules without relying on changes in the expression level of *SlWUS*, *SlCLV3*, and *SlTAG1*.

### 3.2. Low Temperature Induced Accumulation of Bioactive GA_1_ and GA_4_

GA metabolites show a strong temperature-dependent accumulation during flower bud differentiation in tomato (Figure 4; Table 3). By analyzing the expression profiles of GAs, the changes in the expression of *SlGA2ox* genes encoding degradation enzymes related to GA metabolism were downregulated significantly after lower-temperature treatment in agreement with a previous report [41]. In order to further elucidate the results of changes in the expression of *SlGA2ox* genes in tomato after lower overnight temperature treatment, the GA content in tomato was analyzed. GA_1_, GA_4_, and GA_7_ were detected at all stages of flower bud differentiation, while GA_3_ was detected at pre-flower bud differentiation (Table 3). GA_1_ and GA_4_ were more sensitive to low overnight temperature than GA_7_, since GA_7_ is not the subject of degradation by GA2 oxidases (Table 3) [29], and lower-temperature treatment can increase GAs content during floral bud differentiation, in agreement with a previous report [41].

### 3.3. Effect of Gibberellins on the Formation of Tomato Locules

Gibberellins (GAs) are a class of plant hormones involved in the regulation of flower development. The GA-deficient *ga1-3* mutant shows retarded growth of all floral organs [42]. In the SAM, gibberellins accumulate in the peripheral zone cells and promote lateral organ initiation and play a vital role in regulating the carpel number [25,43]. Exogenous gibberellins promoted an increase in tomato locule number as previously reported (Figure 6) [25]. In rice, *GA2ox1* was expressed in the rib meristem, in which it restricts access of bioactive GAs to the SAM [44]. Consistent with this, expression at the shoot apex comparable to that in rice was reported in *Arabidopsis* [45]. Furthermore, in *Arabidopsis*, the number of cells in the root meristem of *GA2ox2*-overexpression lines was lower than in the wild type, and *GA2ox2* overexpression suppressed root elongation [46]. Our analysis revealed that tomato locule number increased at lower night temperature, and the lower night temperature downregulated the expression level of most *SlGA2ox* genes (Figure 4 and Figure 5). Thus, our study illustrates that lower night temperature can reduce the expression of the *SlGA2ox* genes (Figure 4), leading to increased bioactive GA_1_ and GA_4_ (Table 3), and promoting carpel development by controlling cell division and eventually increasing tomato locule number. Based on the data presented in this work, we propose a pathway that may be involved in temperature-regulated locule number. Under cold stress, *SlGA2ox* genes were downregulated, leading to GA_1_ and GA_4_ accumulation, thereby increasing the locule number of tomato.

## 4. Materials and Methods

### 4.1. Plant Materials, Growth Conditions, and Treatments

Tomato multi-locule “MLK1” line, which produces large fruit with 10 or more locules, was grown in a greenhouse (average day/night temperatures, 28 °C/15 °C) with natural light and a relative humidity of 60% [23]. When the tomato seedlings had 2–3 true leaves (pre-flower bud differentiation), they were moved to three growth rooms (KLAN-03, kooland, Beijing, China) and kept at three different overnight temperatures: 10, 15, and 20 °C (average daily temperature: 28 °C). The three groups were subjected to identical daily natural light conditions (400 µmol·m^−2^·s^−1^) with 60% relative humidity. For each treatment, shoot apexes, each approximately 5 mm, were removed from plants for RNA sequencing at time 0 days (control, pre-flower bud differentiation), after 10 days (T10-d10, T15-d10, and T20-d10; sepal/petal primordium formation), and after 20 days (T10-d20, T15-d20, and T20-d20; carpel primordium formation). After the treatments, 10 seedlings from each treatment were moved to the greenhouse, and the number of locules was investigated.

Chemical treatments with GA_1_ (100 µM; TRC, Toronto, ON, Canada), GA_4_ (100 µM; TRC, Canada), PAC (100 µM), and H_2_O as a control were realized on seedlings with 2–3 true leaves every two days for a total of three times per treatment.

### 4.2. Phenotypic Analysis of Tomato Fruit

We investigated the four fruit locule numbers of the first inflorescences from 10 tomato plants at different overnight temperatures and hormone treatments. And the fruit morphology of the first inflorescence was observed and recorded. All values were expressed as the mean ± standard deviation, using SPSS 22 software. The average values were further subjected to analysis of significant differences by Duncan’s multiple range test. A value of *p* < 0.05 was considered as statistically significant.

### 4.3. RNA Extraction and Transcriptome Sequencing

RNA extraction, sequencing library construction, and Illumina sequencing were performed by GENEWIZ, China. Total RNA of the seven samples (two biological replications per sample) was extracted using TRIzol Reagent (Invitrogen, Carlsbad, CA, USA). Total RNA of each sample was quantified and qualified by Agilent 2100 Bioanalyzer (Agilent Technologies, Palo Alto, CA, USA), NanoDrop (Thermo Fisher Scientific Inc., USA), and 1% agarose gel. Next, a sequencing library was constructed with an average insert size of 360 bp following the manufacturer’s protocol (NEBNext^®^ Ultra™ RNA Library Prep Kit for Illumina^®^) and sequenced using an Illumina HiSeq instrument (Illumina, San Diego, CA, USA).

### 4.4. Data Analysis

In order to remove low-quality sequences, including adapters, sequences shorter than 20 bases, polymerase chain reaction primers, or fragments, and pass-filter data in fasta format were excluded using Trimmomatic (v0.30). The resulting clean data were aligned to the tomato reference genome via Hisat2 (v2.0.1) software [47]. Then, the reads were uniquely mapped to the annotated tomato reference genes, and isoform expression levels from the pair-end clean data were estimated using HTSeq (v0.6.1). Differential expression analysis was conducted using the DESeq Bioconductor package, a model based on negative binomial distribution [48]. After adjustment using Benjamini and Hochberg’s approach for controlling the false discovery rate (FDR), we used FDR ≤0.05 and the absolute value of log2 (Ratio) ≥1 as the thresholds to judge the significance of gene expression difference.

GO Term Finder was used to determine the Gene Ontology (GO) terms that annotated a list of enriched genes with a significant *p*-value less than 0.05. We used in-house scripts to study significant DEGs in Kyoto Encyclopedia of Genes and Genomes (KEGG) pathways (http://en.wikipedia.org/wiki/KEGG).

### 4.5. qRT–PCR Analysis

Total RNA was extracted using an RNA prep Pure Plant total extraction kit (Tiangen Biotech, Beijing, China). Then, RNA samples were reverse-transcribed into cDNAs using PrimeScript^TM^ RT Master Mix (Takara, Dalian, China). Real-time PCR analysis was performed using SYBR Green PCR Master Mix (Tiangen, Beijing, China), and using the ABI 7500 Real-Time PCR system and Software 7500 ver. 2.0.3 (Applied Biosystems, USA) with three replications. Primers utilized in this study were designed using Primer 5.0 software (Premier Biosoft, USA, Figure S1). Data were analyzed using SPSS 22 Software (SPSS Inc., USA).

### 4.6. Quantification of Phytohormones

About 1 g of shoot apex was sampled from CK, T10-d10, T20-d10, T10-d20, and T20-d20, with three biological replicates, and was subjected to analysis. Phytohormones were extracted from samples using 2-propanol–H_2_O–concentrated HCl (2:1:0.002, *v*/*v*/*v*) extraction. Phytohormones were quantified as described by Pan et al. with some modifications using a liquid chromatography–mass chromatography system (Agilent 1290, AB company Qtrap6500; California, CA, USA) [49].

## Figures and Tables

**Table 1:** Read number, based on RNA sequencing (RNA-Seq) data in tomato.

Samples	Total Raw Reads	Total Reads	Total Mapped (%)	Uniquely Mapped (%)
CK	73,023,120	72,347,574	67,829,267 (93.75)	65,060,425 (89.93)
T10-d10	64,078,977	63,600,058	60,112,344 (94.52)	57,752,715 (90.81)
T15-d10	53,299,090	52,897,547	49,928,204 (94.39)	47,913,998 (90.58)
T20-d10	52,736,947	52,392,032	49,551,460 (94.58)	47,523,527 (90.71)
T10-d20	59,988,215	59,600,279	56,433,514 (94.69)	54,114,303 (90.80)
T15-d20	53,972,546	53,397,506	49,836,432 (93.33)	47,801,791 (89.52)
T20-d20	67,265,972	66,417,818	62,130,622 (93.55)	59,583,787 (89.71)

**Table 2:** KEGG pathway enrichment analysis among differentially expressed genes (DEGs). ID—identifier.

Pathway ID	Pathway	Number of DEGs with Pathway Annotation					
		T10-d10 vs. CK	T15-d10 vs. CK	T20-d10 vs. CK	T10-d20 vs. CK	T15-d20 vs. CK	T20-d20 vs. CK
ko04075	Plant hormone signal transduction	0	0	38	58	44	48
ko00500	Starch and sucrose metabolism	4	6	0	37	33	31
ko04626	Plant-pathogen interaction	0	0	0	34	24	27
ko00360	Phenylalanine metabolism	0	0	0	31	0	0
ko00520	Amino sugar and nucleotide sugar metabolism	0	3	0	23	20	18
ko00940	Phenylpropanoid biosynthesis	5	5	0	10	29	32
ko00941	Flavonoid biosynthesis	1	0	7	15	11	12
ko00904	Diterpenoid biosynthesis	1	4	7	15	13	13
ko00592	Alpha-linolenic acid metabolism	0	0	0	15	9	11
ko00910	Nitrogen metabolism	1	2	0	12	10	10

**Table 3:** Levels of gibberellins (GAs) in the different treatments.

	CK	T10-d10	T20-d10	T10-d20	T20-d20
**GA**					
**Bioactive GAs**					
GA_1_	0.010 ± 0.002 ^d^	0.306 ± 0.048 ^b^	0.087 ± 0.061 ^cd^	0.511 ± 0.055 ^a^	0.183 ± 0.017 ^c^
GA_3_	0.216 ± 0.018	ND	ND	ND	ND
GA_4_	0.078 ± 0.013 ^b^	0.058 ± 0.011 ^b^	0.074 ± 0.011 ^b^	0.123 ± 0.028 ^a^	0.049 ± 0.010 ^b^
GA_7_	0.153 ± 0.024 ^a^	0.054 ± 0.001 ^b^	0.041 ± 0.013 ^b^	0.057 ± 0.011 ^b^	0.033 ± 0.015 ^b^
**Precursors**					
GA_12_	ND	ND	ND	ND	ND
GA_53_	1.901 ± 0.258 ^a^	1.829 ± 0.241 ^a^	1.435 ± 0.163 ^b^	2.079 ± 0.073 ^a^	1.940 ± 0.145 ^a^
GA_20_	0.038 ± 0.014 ^c^	0.051 ± 0.021 ^c^	0.068 ± 0.007 ^c^	0.271 ± 0.025 ^a^	0.164 ± 0.021 ^b^
GA_24_	0.100 ± 0.029 ^b^	0.026 ± 0.005 ^c^	0.019 ± 0.008 ^c^	0.160 ± 0.014 ^a^	0.044 ± 0.017 ^c^
GA_9_	0.093 ± 0.077 ^ab^	0.151 ± 0.028 ^a^	0.096 ± 0.052 ^ab^	0.052 ± 0.003 ^ab^	0.032 ± 0.006 ^b^
**Deactivated GAs**					
GA_8_	0.178 ± 0.032 ^d^	0.965 ± 0.050 ^b^	1.087 ± 0.126 ^ab^	1.194 ± 0.098 ^a^	0.642 ± 0.018 ^c^
GA_29_	ND	ND	ND	0.950 ± 0.240	ND
GA_51_	1.020 ± 0.128 ^a^	0.501 ± 0.113 ^b^	0.436 ± 0.002 ^b^	0.244 ± 0.012 ^c^	0.071 ± 0.026 ^c^

^1^ Note: ND, not detected. ^2^ Endogenous phytohormone levels (ng·g^−1^ fresh weight (FW)) in each plant part. ^3^ Different lowercase letters represent significant differences (*p* < 0.05, Duncan’s multiple range test).

## Supplementary Materials

Supplementary materials can be found at https://www.mdpi.com/1422-0067/20/12/3042/s1: Figure S1. Quantitative real-time PCR primers; Table S1. The raw data of locule number investigation; Table S2. The DEGs identified in different treatments; Table S3. The Gene GO enrichment analysis of the DEGs in different treatments; Table S4. KEGG pathway enrichment analysis of the DEGs in different treatments; Table S5. Expression level and fold change of genes in RNA-seq; Table S6. The raw data of qRT-PCR validation.
