# Supplementary material for: Low Overnight Temperature-Induced Gibberellin Accumulation Increases Locule Number in Tomato
Source: Int J Mol Sci. 2019 Jun 21;20(12):3042. doi: 10.3390/ijms20123042 (PMC6627202; doi:10.3390/ijms20123042)
Supplement: Supplementary file 1 [file ijms-20-03042-s001.zip › Supplemental Figure S1.docx]

**Supplemental Figure S1. Quantitative real-time PCR primers.**

| Gene ID | Gene Name |  | Primer (5’-3’) |
| --- | --- | --- | --- |
| Solyc03g078400 | *SlACTIN* | F | TGTCCCTATTTACGAGGGTTATGC |
|  |  | R | AGTTAAATCACGACCAGCAAGAT |
| Solyc10g007860 | *SlKAO* | F | TTGCTGGTTTTGAATCTGTTGC |
|  |  | R | CCTCTTGTTCCTCTTTAGCTTTGT |
| Solyc02g070430 | *SlGA3ox1* | F | GCTCGCTCTCCTGATGGTGT |
|  |  | R | ATTAATCTTCCTGCTAGCTTTTCC |
| Solyc03g119910 | *SlGA3ox2* | F | TAGACCAAAGGAACCCTCAAATG |
|  |  | R | AGGAGCACAAAGCCGAACAG |
| Solyc07g056670 | *SlGA2ox2* | F | TTCTTCTCCTCTCCCCTCTCTG |
|  |  | R | GCCATTTGTTCCAATTTGTTTATT |
| Solyc07g061720 | *SlGA2ox4* | F | *CCTGCCATTACTCATCACTTCCT* |
|  |  | R | *GGCCCTGCCTTTAGTTTCTC* |
| Solyc07g061730 | *SlGA2ox5* | F | TCTTCTCCTCTCCCCTCTCTGA |
|  |  | R | GATATCGCCACTTTGTCCGAT |
| Solyc02g083950 | *SlWUS* | F | CCAGCAACTTACCCTTTTCTTG |
|  |  | R | TAAAGCAGAGTTACCCCTTTGG |
| Solyc11g071380 | *SlCLV3* | F | AAAGGAAGTTGCTCCTGTGAA |
|  |  | R | CCTCTTAGCTCCCAATCAGC |
| Solyc02g071730 | *SlTAG1* | F | CTTGATGCCAGGGAGTTCAT |
|  |  | R | ATCGAATTGCTGAGGTGGAG |
